# Highly Crosslinked Polyethylene Tibial Post Fracture in the Unafflicted Limb of a Patient with Unilateral Lower Limb Poliomyelitis: A Case Report

**DOI:** 10.5704/MOJ.1903.008

**Published:** 2019-03

**Authors:** WC Lee, LHJ Wee

**Affiliations:** Department of Orthopaedic Surgery, Tan Tock Seng Hospital, Singapore

**Keywords:** highly crosslinked polyethylene, posterior-stabilised total knee arthroplasty, poliomyelitis, tibial post fracture

## Abstract

We present a unique case of tibial post fracture of a posterior-stabilised total knee arthroplasty (PS-TKA) using highly crosslinked polyethylene (HXLPE) in the unafflicted limb of a patient who had poliomyelitis. The tibial post is an upright structure perpendicular to the PE insert articular surface which articulates with the cam of the femoral component to prevent excessive posterior translation of the tibia. We explore the choice of PS polyethylene (PE) inserts in patients with neuromuscular disorders (NMD). A 74-year old gentleman presented with recurrent knee pain seven years after the index PS-TKA with HXLPE. The TKA was performed on the unafflicted left limb (contralateral to the weak side affected by poliomyelitis). The posterior drawer test was positive. During the single-stage revision surgery, the HXLPE tibial post was noted to be broken. The liner was replaced with a thicker non-HXLPE. The patient achieved an excellent outcome at one-year post-surgery. This is the first report of HXLPE tibial post fracture in the unaffected knee of a patient with NMD affecting the lower limb. The HXLPE’s reduced resistance to fatigue crack propagation might not be suitable in PS-TKA where there might be focal stress points on the tibial post, which was amplified in this case as it was the limb that the patient most depended on. When managing end-stage osteoarthritis with TKA in the unafflicted knee of a patient with NMD causing lower limb weakness, the selection of polyethylene material in PS-TKA may need more consideration than previously thought.

## Introduction

The posterior-stabilised total knee arthroplasty (PS-TKA) has an uncommon but significant complication unique to its design-fracture of the tibial post of the polyethylene (PE) insert. The tibial post is an upright structure perpendicular to the PE insert articular surface which articulates with the cam of the femoral component to prevent excessive posterior translation of the tibia. We describe a patient with poliomyelitis who developed a tibial post fracture of the highly crosslinked PE (HXLPE) articular insert following PS-TKA in his normal unafflicted limb.

## Case Report

A 74-year old gentleman who underwent a left PS-TKA seven years previously defaulted his follow-up appointments after two years as he was satisfied with the TKA. He was symptom free until his current presentation of acute pain while getting out of a chair. Subsequently, the pain recurred mostly during climbing stairs. He had a history of poliomyelitis, which resulted in severe right lower limb weakness, but spared his left lower limb.

On physical examination, the left knee had a well-healed midline longitudinal scar. There was a small effusion with mild warmth, but without erythema. The range of motion (ROM) was from -3º to 110º with audible clicks when ranging the knee, compared to his last recorded ROM of 0º to 120º. There was a posterior sag sign, and the posterior drawer test was positive with no end point. His body mass index was 24.4 and he walked using a walking frame. The examination of his hips and spine were unremarkable.

**Fig. 1:**
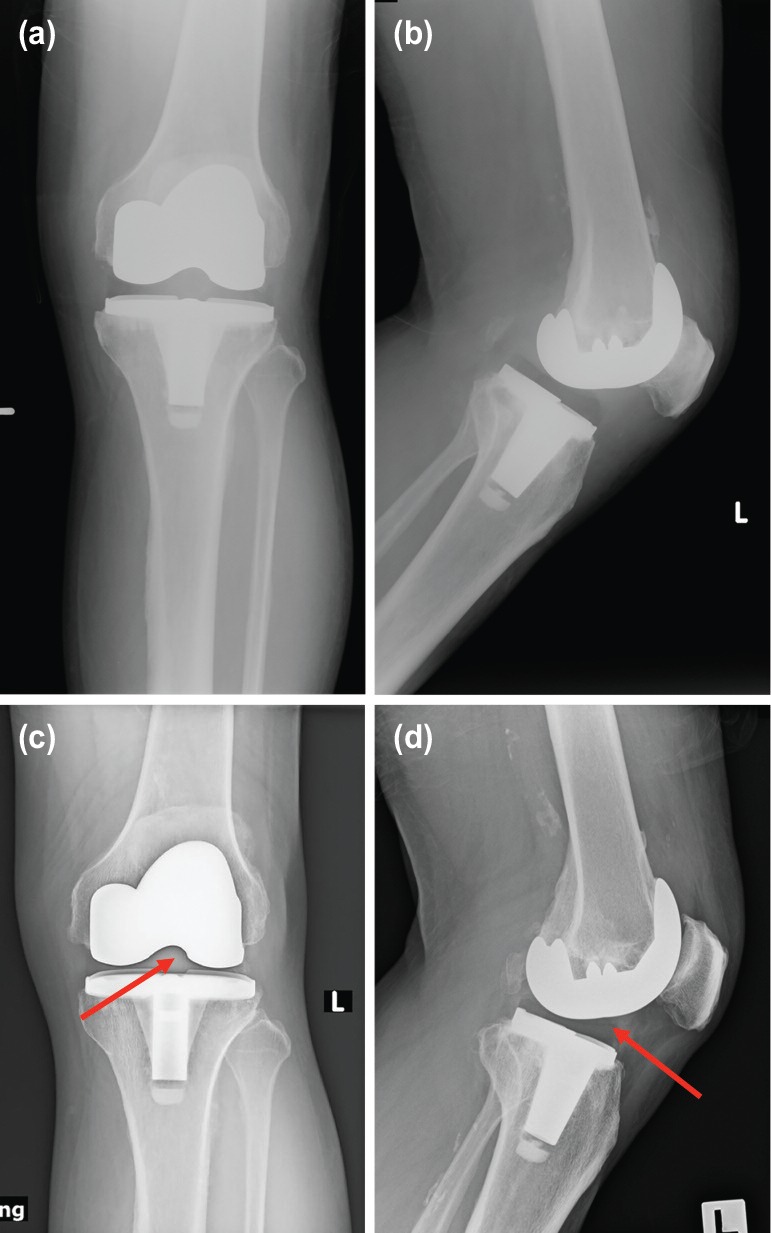
Plain radiographs of the total knee arthroplasty (TKA) at various time points. (a) Anteroposterior (AP) view after the index surgery. (b) Lateral view after the index surgery. (c) AP view on presentation. Arrow denotes the likely position of the tibial post of the polyethylene (PE) insert. (d) Lateral view on presentation. Arrow denotes the likely position of the tibial post of the PE insert.

Plain radiographs after the index TKA and on presentation (Fig. 1), as well as a computed tomography (CT) scan of the knee showed that the TKA had satisfactory alignment and acceptable femoral component positioning without any signs of loosening. The total white cell count, erythrocyte sedimentation rate and C-reactive protein levels were not elevated.

**Fig. 2:**
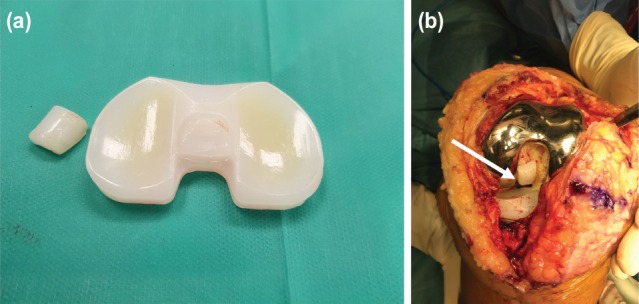
(a) Fracture of the tibial post at its base. (b) Intra-operative image of the broken post in the femoral notch, denoted by the white arrow.

The impression was that of posterior instability, likely due to tibial post failure. The patient consented for a revision TKA. The index PS-TKA comprised Zimmer NexGen LPS [Zimmer, Warsaw, IN] cemented femoral and tibial components, with a 12mm thick PS HXLPE insert. Intraoperatively, the only significant finding was that the HXLPE tibial post had fractured at its base (Fig. 2a) and was freely floating within the notch of femoral component (Fig. 2b). In the setting of balanced gaps, stable and well-positioned existing TKA components, and the absence of infection, the liner was exchanged with a 17mm-thick non-HXLPE insert. Intra-operative testing confirmed good stability and ROM (0º to 120º with no recurvatum).

At one-year post-operation, the knee pain had resolved and he was ambulant with a walking stick. His knee flexion range was from 0º to 120º and was stable. He had marked improvement in his Oxford Knee Score (42 from 27) and Knee Society Clinical Rating Knee Score (153 from 90).

## Discussion

The occurrence of tibial post fractures involving HXLPE is rare. Furthermore, this case is unique as it involves the functionally normal limb of a patient with poliomyelitis causing significant contralateral lower limb weakness. Hence, it merits report so that more data on indications and limitations may be gathered^1^. Herein, we discuss the choice of PE insert in patients with similar neuromuscular disorders (NMD) causing asymmetrical lower limb weakness.

In a patient with poliomyelitis and similar NMD, the unafflicted knee is subjected to greater compressive and shear stresses as the patient attempts to compensate for the contralateral limb weakness and instability during ambulation. Furthermore, the stance phase in the unafflicted limb is prolonged, subjecting the knee to more loading stresses^2^. However, TKA has been shown to provide good long-term outcomes in young patients leading active lifestyles. Meftah *et al* found that, at a mean follow-up of 12 years, a good proportion of patients were able to participate in recreational activities including gym workout with no mechanical failure^3^. In our patient, an earlier-than-expected failure warrants a review of the suitability of HXLPE for a high-demand knee.

HXLPE confers better wear performance compared to conventional PE, but it comes with the drawback of reduced resistance to fatigue crack propagation^1^. In contrast to total hip arthroplasty, in which HXLPE performed well, the PS-TKA design has a focal point of stress on the tibial post. The contact stress may be increased in situations of greater flexion or hyperextension^1^. Though it is not possible to accurately determine the direction of the tibial post fracture in our case, there would be impingement between the anterior post and the femoral component in extension, and between the femoral cam and the posterior post in flexion. Furthermore, the double radius and the curve-on-flat design of NexGen LPS prostheses results in less constraint during knee flexion and is thought to cause more stress on the post^4^. These stresses were likely to be amplified in our patient as this was his only functional lower limb, on which he was reliant for ambulation.

Our patient’s genu-recurvatum had likely occurred after the tibial post fracture. We posit that the consequent altered gait mechanics and joint stresses led to excessive soft tissue stretching, particularly involving the posterior knee capsule and collateral ligaments. Overloading of the knee over the past seven years owing to polio affecting the contralateral limb could have also contributed to overstretching of the ligaments and capsule. This would explain why increasing the PE thickness during the revision surgery was required to optimise knee stability.

This case report highlights that caution should be exercised when choosing the PE material in patients with NMD. Added weight is given to this when considering Diamond *et al* series involving five cases of PE post fractures in 955 HXLPE PS-TKA cases, compared to none when non-HXLPE PS-TKA was performed in 2566 cases^1^. Nonetheless, larger studies will be needed to further establish the role of HXLPE inserts in patients with NMD.

In summary, when managing end-stage osteoarthritis with TKA in the unafflicted knee of a patient with NMD causing lower limb weakness, the selection of PE material in PS-TKA may need more consideration than previously thought.
